# Impact of Prescription Drug Monitoring Program Implementation on Rates and Characteristics of People Seeing Multiple Prescribers in Primary Care: A Controlled Interrupted Time‐Series Analysis

**DOI:** 10.5694/mja2.70219

**Published:** 2026-06-04

**Authors:** Louisa Picco, Monica Jung, Grant Russell, Samanta Lalic, Mahbod A. Fini, Dan I. Lubman, Rachelle Buchbinder, Ting Xia, Suzanne Nielsen

**Affiliations:** ^1^ Monash Addiction Research Centre (MARC) Eastern Health Clinical School, Monash University Victoria Australia; ^2^ Department of General Practice, School of Public Health and Preventive Medicine Monash University Melbourne Victoria Australia; ^3^ Pharmacy Department Monash Health Melbourne Victoria Australia; ^4^ Turning Point Eastern Health Melbourne Victoria Australia; ^5^ Musculoskeletal Health and Wiser Health Care Units, School of Public Health and Preventive Medicine Monash University Melbourne Victoria Australia

**Keywords:** drugs and alcohol, policy, prescription drug misuse, prescription drugs, primary care

## Abstract

**Objective:**

To examine changes in rates of primary care patients seeing multiple prescribers and characteristics of patients who ceased seeing multiple prescribers for monitored medicines after voluntary implementation of the Victorian prescription drug monitoring program (PDMP).

**Study Design:**

Controlled interrupted time series analysis of primary care electronic medical records.

**Setting:**

A total of 562 general practices across three Victorian healthcare networks (Monash Health, Peninsula Health, Eastern Health).

**Patients:**

People prescribed at least one PDMP‐monitored medicine (e.g., opioids, benzodiazepines) and/or non‐monitored psychotropic medicines (e.g., antidepressants, antipsychotics) between 1 January 2017 and 30 June 2023.

**Intervention:**

Voluntary (1 April 2019) and mandatory (1 April 2020) implementation of the Victorian PDMP.

**Main Outcome Measures:**

Changes in the monthly rate of people seeing multiple prescribers (defined as four or more prescribers) following PDMP implementation for monitored medicines, with non‐monitored medicines used as a control; characteristics of people who ceased seeing multiple prescribers for monitored medicines following PDMP implementation.

**Results:**

Following voluntary PDMP implementation (1 April 2019), there was a significant reduction in the differential step and trend changes in the rates of seeing multiple prescribers between people prescribed monitored and non‐monitored medicines (differential step change: *β*, −3.55 [95% confidence interval (CI), −5.08 to −2.03]; differential trend change: *β*, −0.29 [95% CI, −0.46 to −0.12]). Following mandatory PDMP implementation (1 April 2020), there was no significant step change difference. However, there was an increase in the differential trend change in the rate of seeing multiple prescribers between those prescribed monitored and non‐monitored medicines (differential trend change: *β*, 0.21 [95% CI, 0.05–0.37]; *p* = 0.009). Logistic regression revealed that older age (95% CI, 1.39–1.75), male gender (95% CI, 1.09–1.25), metropolitan residence (95% CI, 1.04 and 1.23) and substance use disorder diagnosis (95% CI, 1.07–1.28) were associated with significantly higher odds of seeing multiple prescribers before PDMP implementation.

**Conclusions:**

Implementation of the PDMP was associated with meaningful reductions in people accessing monitored medicines from four or more prescribers.

## Plain Language Summary

**The Known:** Obtaining high‐risk medicines from multiple prescribers is associated with negative health outcomes, including dependence, overdose and death. The implementation of prescription drug monitoring programs allows prescribers to identify when this high‐risk behaviour is occurring.

**The New:** We demonstrated that implementation of the Victorian prescription drug monitoring program was associated with meaningful reductions in people obtaining monitored medications from four or more prescribers.

**The Implications:** Prescription drug monitoring programs allow prescribers to identify patients obtaining monitored medications from multiple prescribers and are an important policy for mitigating medication‐related harms.

## 1 Introduction

Seeing multiple prescribers for high‐risk medicines (sometimes referred to as ‘doctor shopping’) is strongly associated with negative health outcomes [1]. Although there is no universal definition for ‘seeing multiple prescribers’, this behaviour centres around obtaining prescriptions from multiple prescribers, often in excess of clinical need [2]. In Australia, this is most commonly operationalised as seeing four or more prescribers within 90 days [3, 4]. Obtaining high‐risk medicines, including opioids, benzodiazepines and stimulants, from multiple prescribers has been associated with reduced continuity of care [5], increased medical expenses [5] and increased risk of harm, including non‐medical use, dependence, overdose and death [5, 6].

One common approach to identifying multiple prescriber episodes is via prescription drug monitoring programs (PDMPs). PDMPs are state‐based electronic databases that track the prescribing and dispensing of high‐risk medicines, allowing clinicians (prescribers and pharmacists) to view patients' prescription histories for monitored medications and help identify patients with high‐risk behaviours, including seeing multiple providers or being on high doses [7]. PDMPs do, however, vary considerably in terms of the medications they monitor, who can access them (e.g., only clinicians in Australia, while in the United States, they can also be accessed by law enforcement) and when they are required to be accessed. For example, some jurisdictions mandate use, whereby clinicians must check the PDMP before prescribing or dispensing monitored medications [8].

In 2018, all Australian states and territories committed to real‐time prescription monitoring, with implementation occurring between 2019 and 2023. Victoria was the first jurisdiction to mandate PDMP use, requiring community‐based prescribers and pharmacists to check the PDMP before prescribing or dispensing monitored medications. While PDMPs are implemented at the jurisdictional level, collectively they aim to reduce multiple provider episodes, identify patients who are at risk of harm due to dependence or non‐medical use of monitored medicines and identify patients who may be diverting medicines [9]. Following implementation of the Victorian PDMP, deaths attributed to monitored medicines and prescription medicine‐related hospitalisations decreased [10], while increased initiation of non‐monitored medicines (including antidepressants, pregabalin and tramadol) and reduced low‐dose opioid prescribing have been observed [11, 12].

In the United States, studies have demonstrated reduced multiple prescriber episodes following PDMP implementation [13, 14], particularly where use is mandated [15]. To our knowledge, no Australian study has explored the association between PDMP implementation and multiple prescriber episodes. To address this gap, the current study aimed to examine changes in rates of primary care patients seeing multiple prescribers and characteristics of patients who ceased seeing multiple prescribers for monitored medicines after voluntary implementation of the Victorian PDMP.

## 2 Methods

### 2.1 Study Design and Setting

This retrospective study used routinely collected administrative primary healthcare data extracted from general practice clinics and used with permission from three primary health networks (PHNs) in Victoria, Australia—Eastern Melbourne Primary Health Network, South Eastern Melbourne Primary Health Network and Gippsland Primary Health Network—which cover about 52% of the Victorian population [16].

Victoria is the second most populous state in Australia [17]. PDMP implementation commenced on 1 April 2019; use was voluntary for the first year, and it became mandatory on 1 April 2020. All community‐based prescribers and pharmacists are required to check the PDMP before prescribing and dispensing monitored medications. The Victorian PDMP is commonly integrated into prescribing and dispensing software and utilises a back‐end computerised algorithm that triggers a notification and alert system. Pop‐up notifications appear in the prescribing or dispensing software, informing clinicians whether there are records that need to be reviewed within the PDMP. Clicking on these pop‐ups will automatically redirect clinicians to the patient's record within the PDMP, providing further information. Red notifications, which signify the greatest risk, are triggered when a patient is prescribed a high dose (> 100 mg oral morphine equivalents), a high‐risk drug combination (e.g., opioid and benzodiazepine), or has seen four or more prescribers, with all alerts being triggered based on prescribing and dispensing in the previous 90 days [3]. This study was reported according to RECORD‐PE (the Reporting of Studies Conducted Using Observational Routinely Collected Health Data Statement for Pharmacoepidemiology), an extension of the STROBE (Strengthening the Reporting of Observational Studies in Epidemiology) and RECORD (Reporting of Studies Conducted Using Observational Routinely Collected Health Data) statements (Table S1) [18].

### 2.2 Data Source

De‐identified patient‐level data were extracted from contributing general practices via the Population Level Analysis and Reporting (POLAR) general practice analytics platform, managed by Outcome Health. The prescribing data, extracted with approval from PHNs, included medical records for about 4.7 million patients from 562 general practices, representing about 55% of general practices within the three PHN regions [19, 20, 21]. Data included the following cohort demographics: age, reported in 5‐year categories; gender, based on socially constructed roles, behaviours and identities (male or female); geographically derived socio‐economic status (measured by the Index of Relative Socio‐economic Advantage and Disadvantage [IRSAD], which is a socio‐economic index based on patient's postcode of residence and ranked on a scale from 1 to 10, with 1 indicating the most disadvantaged) [22]; geographic remoteness (measured by Modified Monash Model categories) [23]; and concession card status. Further details of the general practice data collected and curated across the POLAR platform have been comprehensively documented elsewhere [24].

### 2.3 Measures

#### 2.3.1 Multiple Prescriber Episodes Definition

The primary outcome was changes in the monthly prevalence rate of patients (per 1000 patients) seeing multiple prescribers for medicines monitored by the Victorian PDMP. A multiple prescriber alert is triggered when an individual obtains any monitored medications (irrespective of medication class) from four or more prescribers within a 90‐day period [3] and was adopted for the current analyses. On each calendar day, individuals were classified as seeing multiple prescribers if they had received prescriptions for monitored medications from four or more distinct prescribers in the preceding 90 days.

Adults aged ≥ 18 years who were prescribed at least one monitored and/or non‐monitored medicine between 1 January 2017 and 30 June 2023 were included. All medicines monitored by the Victorian PDMP were included in these analyses [3] (Table S2). Non‐monitored psychotropic medications, including antidepressants, antipsychotics, gabapentinoids, tramadol and other psychotropic medicines, were selected, controlling for potential underlying trends that were unlikely to be affected by PDMP implementation. These medications were chosen as they are predominantly used by the same patient population but were not monitored by the PDMP (Table S3). Tramadol, gabapentin and pregabalin were only monitored by the Victorian PDMP from July 2023, which was outside the study's data period [25].

The prescribing clinician was identified by a unique prescriber identifier within that clinic by linking each prescription record to the corresponding activity data recorded on the same date. As the prescriber information was absent from the prescription data but included in the patients' activity data, we matched the patient appointment date (via the clinical activity data) with the date of the prescription data to confirm the prescriber associated with each prescription episode. Overall, 88.3% of the prescription episodes for monitored medicines and 90.8% for non‐monitored medicines were matched to an activity record.

#### 2.3.2 Comorbidities

To examine the changes in rates of patients seeing multiple prescribers, and characteristics of people who ceased to see multiple prescribers, following PDMP implementation, the following comorbidities were examined based on prior literature [26, 27]: (i) mental health diagnosis; (ii) substance use disorder; (iii) headache/migraine; and (iv) chronic pain. These comorbidities were chosen because they are associated with the prevalent use of monitored medications [28, 29]. Data on diagnoses coded using Systematized Nomenclature of Medicine (SNOMED) were used to determine the comorbidities adapted from categorisations used in prior literature (Tables S4.1–S4.4) [27].

### 2.4 Analysis

The monthly prevalence rates of seeing multiple prescribers for monitored and non‐monitored medicines were estimated using a rolling 90‐day window. For each calendar month, we summed the number of individuals who had seen multiple prescribers. The monthly rate was calculated by dividing the number of individuals who, in each month of the study period, received prescriptions from four or more prescribers for monitored medicines in the previous 90 days, by the total number of individuals prescribed monitored medicines in that same month. The same method was used to calculate the rate of seeing multiple prescribers for non‐monitored medications. Results were reported as the number of patients per 1000 people of the relevant population. Data related to three periods: 24 months before voluntary PDMP implementation (1 April 2017 to 31 March 2019); 12 months after voluntary PDMP implementation (1 April 2019 to 31 March 2020); and 39 months after mandatory PDMP implementation (1 April 2020 to 30 June 2023).

Controlling for baseline levels and underlying secular trends [30], controlled interrupted time series analysis was employed to assess changes in the monthly rates of seeing multiple prescribers for both monitored and non‐monitored medicines before voluntary PDMP implementation (before 1 April 2019), during voluntary PDMP use (between 1 April 2019 and 31 March 2020) and after mandatory PDMP implementation (after 1 April 2020). In line with interrupted time series assumptions, we assessed stationarity, autocorrelation, seasonality and potential effects of coronavirus disease 2019 (COVID‐19) pandemic lockdowns. Full details can be found in the Supporting Information (Appendix S1).

We utilised logistic regression analysis to investigate demographics (e.g., age, gender, socio‐economic status) and pre‐existing comorbidities (e.g., mental health and chronic pain conditions) associated with people who ceased seeing multiple prescribers for monitored medications after voluntary PDMP implementation. The independent variable, seeing multiple prescribers for monitored medicines before 1 April 2019 only, was coded as ‘1’, while seeing multiple prescribers for monitored medicines in both periods or only after PDMP implementation was coded as ‘0’. The models were adjusted for relevant covariates, including age, gender and comorbidities. *p* values of less than 0.05 were deemed statistically significant. Analyses were performed using Stata statistical software, release 17 (StataCorp LLC, College Station, TX).

### 2.5 Ethics Statement

Ethics approval was obtained from the Monash Health Human Research Ethics Committee (ID 76744), Monash Health (RES‐22‐0000‐026A), Peninsula Health (SSA/76744/PH‐2022) and Eastern Health (S22‐032‐76744).

## 3 Results

A total of 6,796,173 prescriptions for monitored medicines (810,092 people) and 3,113,184 prescriptions for non‐monitored medicines (366,081 people) were analysed. Almost all the prescriptions were associated with activities by general practitioners (99.9%). During this period, 2.3% (18,474/810,092 people) engaged in seeing multiple prescribers for monitored medicines, and 85.2% of these multiple prescriber episodes (120,846/141,778 episodes) involved at least one opioid. Further investigation of the data revealed that 96% of multiple prescriber episodes occurred within the same clinic.

### 3.1 Multiple Prescriber Trends

**FIGURE 1:**
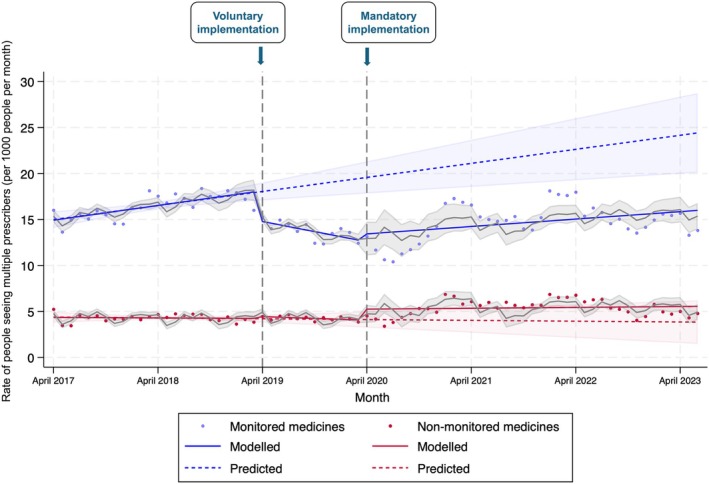
Monthly rate of people receiving prescriptions from four or more prescribers in a given 90 days, per 1000 people. Shaded areas indicate the 95% confidence intervals for the modelled and predicted estimates. Voluntary implementation of the prescription drug monitoring program took effect on 1 April 2019, whereas mandatory implementation took effect on 1 April 2020.

Before voluntary PDMP implementation, there were significant step change (*β*, 10.6 [95% CI, 9.84–11.31]) and trend change (*β*, 0.14 [95% CI, 0.06–0.22]) differences in multiple prescriber rates between people prescribed monitored and non‐monitored medicines (Figure 1, Table 1). Following voluntary PDMP implementation, there was a significant reduction in both the differential step and trend change in multiple prescriber rates between people prescribed monitored and non‐monitored medicines. Relative to the rate of seeing multiple prescribers for non‐monitored medicines, there were 3.55 fewer people per 1000 (95% CI, −5.08 to −2.03) seeing multiple prescribers for monitored medicines immediately following voluntary PDMP implementation, representing a 15.4% reduction, relative to the rates predicted in the absence of PDMP implementation. In the 12 months following voluntary PDMP implementation, there was also a decrease in the rate of people seeing multiple prescribers (*β*, −0.29 [95% CI, −0.46 to −0.12]) relative to the rate with non‐monitored medicines.

**TABLE 1:** Step and trend change in the rate per 1000 people seeing multiple prescribers^a^ for prescription drug monitoring program (PDMP) monitored and non‐monitored medicines following voluntary (April 2019) and mandatory (April 2020) PDMP implementation.

	Estimate (95% confidence interval)	*p*
**Pre‐intervention period (before 1 April 2019)**		
Initial step/level (monitored medicines)	15.73 (14.20 to 17.22)	< 0.001
Initial step/level (non‐monitored medicines)	5.13 (4.36 to 5.91)	< 0.001
Pre‐existing difference in step/level for monitored vs. non‐monitored medicines	10.6 (9.84 to 11.31)	< 0.001
Pre‐existing change (monitored medicines)	0.13 (0.00 to 0.25)	< 0.001
Pre‐existing change (non‐monitored medicines)	−0.01 (−0.06 to 0.03)	0.524
Pre‐existing difference in change for monitored vs. non‐monitored medicines	0.14 (0.06 to 0.22)	0.001
**Voluntary PDMP implementation period (from 1 April 2019)**		
Step change (monitored medicines)	−3.04 (−5.34 to −0.75)	0.001
Step change (non‐monitored medicines)	0.51 (−0.41 to 1.43)	0.274
Differential step change in the rate of seeing multiple prescribers for monitored vs. non‐monitored medicines	−3.55 (−4.93 to −2.18)	< 0.001
Change (monitored medicines)	−0.34 (−0.60 to −0.09)	< 0.001
Change (non‐monitored medicines)	−0.05 (−0.17 to 0.06)	0.367
Differential change in the rate of seeing multiple prescribers for monitored vs. non‐monitored medicines	−0.29 (−0.43 to −0.15)	< 0.001
**Mandatory PDMP implementation period (from 1 April 2020)**		
Step change (monitored medicines)	2.03 (−1.39 to 5.45)	0.068
Step change (non‐monitored medicines)	2.38 (1.12 to 3.63)	< 0.001
Differential step change in the rate of seeing multiple prescribers for monitored vs. non‐monitored medicines	−0.35 (−2.51 to 1.82)	0.753
Change (monitored medicines)	0.25 (0.00 to 0.52)	< 0.001
Change (non‐monitored medicines)	0.04 (−0.08 to 0.17)	0.496
Differential change in the rate of seeing multiple prescribers for monitored vs. non‐monitored medicines	0.21 (0.08 to 0.35)	0.002

^a^ Seeing multiple prescribers was defined as receiving prescriptions for monitored medications from four or more prescribers in the previous 90 days.

Following mandatory PDMP implementation, there were no significant differences in the step change in seeing multiple prescriber rates between people prescribed monitored and non‐monitored medicines; however, there was an increase over time in the differential change in the rate of seeing multiple prescribers in those prescribed monitored medications, relative to the non‐monitored medicines rate (differential trend change: *β*, 0.21 [95% CI, 0.05–0.37]; *p* = 0.009).

### 3.2 Characteristics Associated With Seeing Multiple Prescribers

Compared with people aged 20–44 years, those who were aged 85 years or older were 1.56 (95% CI, 1.39–1.75) times as likely to see multiple prescribers for monitored medications before PDMP implementation. Males, people whose gender was coded as missing/unspecified in the data and people living in metropolitan areas (compared with rural and remote areas) were 1.17 (95% CI, 1.09–1.25), 4.80 (95% CI, 3.53–6.52) and 1.13 (95% CI, 1.04–1.23) times more likely to see multiple prescribers before PDMP implementation, respectively. A substance use disorder diagnosis was also significantly associated with higher odds of seeing multiple prescribers before PDMP implementation (odds ratio [OR], 1.17 [95% CI, 1.07–1.28]) (Table 2).

**TABLE 2:** Comparison of demographic characteristics and comorbidities of people who engaged in seeing multiple prescribers for monitored medicines only before prescription drug monitoring program (PDMP) implementation (1 April 2019) versus people who engaged in seeing multiple prescribers both before and after or only after PDMP implementation.

	Number (%) of people who engaged in seeing multiple prescribers for monitored medicines only before PDMP implementation (1 April 2019) (*N* = 4258)	Number (%) of people who engaged in seeing multiple prescribers for monitored medicines before and after or only after PDMP implementation (reference group) (*N* = 14,216)	Odds ratio (95% confidence interval)^a^
**Demographic characteristics**			
Age (years)^b^			
20–44	1050 (24.7%)	4007 (28.2%)	Reference
45–64	1493 (35.1%)	4823 (33.9%)	**1.18 (1.08–1.29)**
65–84	1124 (26.4%)	3938 (27.7%)	1.09 (0.99–1.20)
≥ 85	591 (13.9%)	1448 (10.2%)	**1.56 (1.39–1.75)**
Gender			
Female	2474 (58.1%)	8928 (62.8%)	Reference
Male	1687 (39.6%)	5215 (36.7%)	**1.17 (1.09–1.25)**
Missing/not specified	97 (2.3%)	73 (0.5%)	**4.80 (3.53–6.52)**
Concessional beneficiary status^c^			
Non‐beneficiary	1474 (34.6%)	4762 (33.5%)	Reference
Beneficiary	2784 (65.4%)	9454 (66.5%)	0.95 (0.89–1.02)
Remoteness^d^			
Metropolitan areas	3196 (75.1%)	10,432 (73.4%)	**1.13 (1.04–1.23)**
Regional centres	216 (5.1%)	670 (4.7%)	1.19 (0.99–1.41)
Rural and remote areas	846 (19.9%)	3114 (21.9%)	Reference
Socio‐economic disadvantage^e^			
1–2, most disadvantaged	894 (21.0%)	2773 (19.5%)	Reference
3–4	502 (11.8%)	1898 (13.4%)	**0.82 (0.72–0.93)**
5–6	1054 (24.8%)	3560 (25.0%)	0.92 (0.83–1.02)
7–8	949 (22.3%)	3352 (23.6%)	**0.88 (0.79–0.97)**
9–10, least disadvantaged	859 (20.2%)	2633 (18.5%)	1.01 (0.91–1.13)
**Comorbidities** ^f^			
Mental health diagnosis	2966 (69.7%)	9959 (70.1%)	0.98 (0.91–1.06)
Substance use disorder	741 (17.4%)	2170 (15.3%)	**1.17 (1.07–1.28)**
Headache/migraine	807 (19.0%)	2904 (20.4%)	**0.91 (0.84–0.99)**
Chronic pain	812 (19.1%)	3256 (22.9%)	**0.79** **(0.73–0.86)**

^a^ Odds ratios and 95% confidence intervals were derived from a logistic regression model adjusted for all covariates listed in the table. Bold values indicate statistical significance at *p* < 0.05.

^b^ Age at cohort entry in 2018.

^c^ Beneficiary includes those who hold one of the following: (i) Commonwealth Seniors Health Card, (ii) Veteran Card (Department of Veterans' Affairs), (iii) Health Care Card, (iv) Pensioner Concession Card. Where beneficiary status was not specified, people were assumed to be non‐beneficiary.

^d^ Remoteness was measured on a scale of Modified Monash Model categories MM1–MM7. The categories were classified into metropolitan areas (MM1), regional centres (MM2), and rural and remote areas (MM3–MM7).

^e^ Geographically derived socio‐economic status was measured by Index of Relative Socio‐economic Advantage and Disadvantage (IRSAD) deciles. IRSAD is ranked on a scale from 1 to 10, with 1 indicating the most disadvantaged.

^f^ Comorbidities were examined as a binary variable.

The odds of seeing multiple prescribers before PDMP implementation were significantly lower for people living in areas of IRSAD deciles 1 or 2 (most disadvantaged, on a scale from 1 to 10) compared with those living in IRSAD deciles 3 or 4 (OR, 0.82 [95% CI, 0.72–0.93]) or 7 or 8 (OR, 0.88 [95% CI, 0.79–0.97]). Migraine or headache (OR, 0.91 [95% CI, 0.84–0.99]) and chronic pain (OR, 0.79 [95% CI, 0.73–0.86]) diagnoses were also associated with significantly lower odds of seeing multiple prescribers before PDMP implementation compared to people without a diagnosis record.

## 4 Discussion

To our knowledge, this is the first Australian study to examine the association between PDMP implementation in Victoria and multiple prescriber rates for monitored medicines. The largest reduction in multiple prescriber rates occurred immediately following voluntary PDMP implementation, with a sustained decline in the longer term. Although these estimates are considered conservative, as not all Victorian practices were included, they do align with concurrent reductions in opioid‐related harms, including emergency department presentations, following PDMP implementation in Victoria [31].

Interestingly, following mandatory PDMP implementation, no further significant reductions in multiple prescriber rates were observed, relative to voluntary use, suggesting that much of the observed change occurred during voluntary use, potentially due to a range of factors. For example, unlike US PDMPs, which were implemented to identify patients with high‐risk behaviours and prescribers undertaking suspected fraudulent prescribing [32], Australian PDMPs focus on identifying patients engaging in high‐risk behaviours [33]. During the 1‐year voluntary implementation period, the focus centred on clinical responses to high‐risk behaviours, which may have provided considerable time for uptake, and may explain the limited changes following mandatory implementation. Impacts relating to COVID‐19 may have also contributed to this finding. Further research is needed to assess other possible contributors, including changing clinician engagement and alert fatigue.

The reduction in multiple prescriber rates following PDMP implementation appeared greatest among people who were older, male and living in metropolitan areas, and those who had a substance use disorder diagnosis. We were unable to identify any comparable studies that examined the impact of PDMP implementation on the characteristics of people seeing multiple prescribers. The current findings are important for clinical practice in several ways. While multiple prescriber episodes commonly occurred within the same clinic, given most general practitioners work within practices with six or more doctors and only about 2% work in solo practices [34], this can still result in fragmented care. When care for an underlying condition or complaint is provided by three or more different general practitioners, it can lead to delayed or missed diagnoses, inappropriate prescribing and failure of preventative medicine [35], and this can occur both within and between clinics. Before PDMP implementation, it was more challenging to quickly identify when multiple prescribers (including those in the same clinic) were prescribing monitored medications in the absence of PDMP alerts. Therefore, multiple prescriber PDMP alerts, if they help reduce this practice, may improve continuity of care, which in turn can enhance patient satisfaction and trust, improve patient outcomes and may ultimately lower mortality rates [36]. Currently, little is known about whether clinicians receiving such alerts can differentiate between possible non‐medical use and those with complex health needs, where multiple prescribers may be clinically appropriate. Studies reporting patient behaviours, including medication diversion, may also help to better understand the clinical outcomes associated with PDMPs.

There is limited understanding of why patients may see multiple prescribers, which may include limited availability of a preferred prescriber or the need to consult prescribers across different specialties. Greater understanding of these drivers may inform strategies to reduce fragmented care, which is known to carry risks for both patients and prescribers [35]. There is also limited guidance for clinicians on how to appropriately care for patients with multiple prescribers [37], and previous research has shown that PDMP use can result in negative or unintended consequences for patients, including stigmatisation, abrupt medication discontinuation and dismissal from practices [1, 37, 38]. The current finding reinforces the importance of supporting clinicians to appropriately respond to PDMP alerts, while avoiding abrupt medication discontinuations, which have been associated with adverse patient outcomes, including transitioning from prescription to illicit opioids, hospitalisation, overdose and death [39].

### 4.1 Limitations

The current study utilised a large primary care dataset, encompassing prescribed medications irrespective of their subsidy status, covering three large and diverse health regions, providing geographic and socio‐economic diversity, and is representative of national opioid use patterns [16, 40]. This is one of the only studies outside the United States to examine the association between PDMP implementation and multiple prescriber rates, broadening our understanding of this widely adopted supply‐side policy. Patients may have seen prescribers outside the three PHNs included in this study or specialists outside of primary care, meaning some multiple prescriber episodes may not have been captured. Therefore, the current findings are likely to be conservative and may preferentially reflect instances when seeing multiple prescribers was not optimal, given specialists were not considered. We were unable to discern cases where seeing multiple prescribers was clinically appropriate or determine possible unintended consequences of PDMP implementation, including obtaining medications from unregistered sources or transitions from prescription to illicit drugs. Individual prescriber information is not directly linked to prescribing episodes, therefore we could not confirm with complete certainty whether same‐day general practitioner activity and prescribing episodes were fully associated, though the high linkage rate suggests that this was the case. Patients and prescribers may have also attended or consulted at multiple clinics, precluding hierarchical clustering at the prescriber or clinic level from being reliably modelled. The heterogeneity of the logistic regression reference group (which included individuals who saw multiple prescribers before and after PDMP implementation and those who saw multiple prescribers only after PDMP) may have introduced selection bias. Finally, restrictions relating to the COVID‐19 pandemic coincided with mandatory PDMP implementation, which may have resulted in fewer people seeing multiple prescribers due to travel restrictions and changes in general practitioners' availability, including face‐to‐face consultations.

## 5 Conclusion

Voluntary PDMP implementation was associated with a large reduction in the number of patients seeing multiple prescribers for monitored medicines. Observed reductions appeared greatest among people who were male, older, living in metropolitan areas and socio‐economically disadvantaged and those who had a documented substance use disorder.

## Author Contributions

**Louisa Picco:** conceptualisation, methodology, writing – original draft, writing – review and editing. **Monica Jung:** conceptualisation, data curation, formal analysis, methodology, writing – review and editing. **Grant Russell:** funding acquisition, methodology, writing – review and editing. **Samanta Lalic:** funding acquisition, methodology, writing – review and editing. **Mahbod A. Fini:** data curation, writing – review and editing. **Dan I. Lubman:** funding acquisition, methodology, writing – review and editing. **Rachelle Buchbinder:** funding acquisition, methodology, writing – review and editing. **Ting Xia:** conceptualisation, data curation, formal analysis, methodology, supervision, writing – review and editing. **Suzanne Nielsen:** conceptualisation, funding acquisition, methodology, project administration, supervision, writing – review and editing.

## Funding

This work was supported by the National Health and Medical Research Council (NHMRC) (GNT2002193). Louisa Picco, Rachelle Buchbinder, Dan I Lubman and Suzanne Nielsen are recipients of NHMRC Investigator Grants (Nos 2016909, 1194483, 1196892 and 2025894). The funder had no role in any of the research‐related activities or the write‐up.

## Disclosure

Not commissioned; externally peer‐reviewed.

## Conflicts of Interest

The authors declare no conflicts of interest.

## Supporting information


**Table S1:** Reporting of studies Conducted using Observational Routinely collected health Data statement for PharmacoEpidemiology (RECORD‐PE) checklist.
**Table S2:** List of medicines monitored by the Victorian Prescription Drug Monitoring Program.
**Table S3:** List of other psychotropic medicines not monitored by the Victorian Prescription Drug Monitoring Program.
**Tables S4.1–S4.4:** List of diagnosis codes.
**Appendix S1:** Model specification.

## Data Availability

This study did not generate original data.
