# Supplementary material for: Impact of Prescription Drug Monitoring Program Implementation on Rates and Characteristics of People Seeing Multiple Prescribers in Primary Care: A Controlled Interrupted Time‐Series Analysis
Source: Med J Aust. 2026 Jun 4;224(6):e70219. doi: 10.5694/mja2.70219 (PMC13238316; doi:10.5694/mja2.70219)
Supplement: Supplementary file 1 — Table S1: Reporting of studies Conducted using Observational Routinely collected health Data statement for PharmacoEpidemiology (RECORD‐PE) checklist. Table S2: List of medicines monitored by the Victorian Prescription Drug Monitoring Program. Table S3: List of other psychotropic medicines not monitored by the Victorian Prescription Drug Monitoring Program. Tables S4.1–S4.4: List of diagnosis codes. Appendix S1: Model specification. [file MJA2-224-0-s001.pdf]

MJA
The Medical Journal of Australia

Supporting Information

Supplementary material

This appendix was part of the submitted manuscript and has been peer reviewed.
It is posted as supplied by the authors.

Appendix to: Picco L, Jung M, Russell GM, Lalic S, Alian M, Lubman DI, Buchbinder R, Xia T, Nielsen S. Impact of Prescription Drug Monitoring Program Implementation on Rates and Characteristics of People Seeing Multiple Prescribers in Primary Care: a Controlled Interrupted Time-Series Analysis. *Med J Aust* 2026; doi: 10.5694/mja2.70219

Supplementary Materials

Table S1. Reporting of studies Conducted using Observational Routinely collected health Data statement for PharmacoEpidemiology (RECORD-PE) checklist

Table S2. List of medicines monitored by the Victorian Prescription Drug Monitoring Program

Table S3. List of other psychotropic medicines not monitored by the Victorian Prescription Drug Monitoring Program

Table S4.1-4.4 List of diagnosis codes

Appendix 1. Model specification

Table S1. The RECORD statement for pharmacoepidemiology (RECORD-PE) checklist of items, extended from the STROBE and RECORD statements, which should be reported in non-interventional pharmacoepidemiological studies using routinely collected health data

Item No	STROBE items	RECORD items	RECORD-PE items	Page No
**Title and abstract**				
1	(a) Indicate the study's design with a commonly used term in the title or the abstract.<br>(b) Provide in the abstract an informative and balanced summary of what was done and what was found.	1.1: The type of data used should be specified in the title or abstract. When possible, the name of the databases used should be included.<br>1.2: If applicable, the geographical region and timeframe within which the study took place should be reported in the title or abstract.<br>1.3: If linkage between databases was conducted for the study, this should be clearly stated in the title or abstract.	—	1
**Introduction**				
Background rationale				
2	Explain the scientific background and rationale for the investigation being reported.	—	—	3
Objectives				
3	State specific objectives, including any prespecified hypotheses.	—	—	4
**Methods**				
Study design				
4	Present key elements of study design early in the paper.	—	4.a: Include details of the specific study design (and its features) and report the use of multiple designs if used.<br>4.b: The use of a diagram(s) is recommended to illustrate key aspects of the study design(s), including exposure, washout, lag and observation periods, and covariate definitions as relevant.	4
Setting				
5	Describe the setting, locations, and relevant dates, including periods of recruitment, exposure, follow-up, and data collection.	—	—	4

Participants				
6	(a) Cohort study—give the eligibility criteria, and the sources and methods of selection of participants. Describe methods of follow-up. Case-control study—give the eligibility criteria, and the sources and methods of case ascertainment and control selection. Give the rationale for the choice of cases and controls. Cross sectional study—give the eligibility criteria, and the sources and methods of selection of participants.<br>(b) Cohort study—for matched studies, give matching criteria and number of exposed and unexposed. Case-control study—for matched studies, give matching criteria and the number of controls per case.	6.1: The methods of study population selection (such as codes or algorithms used to identify participants) should be listed in detail. If this is not possible, an explanation should be provided.<br>6.2: Any validation studies of the codes or algorithms used to select the population should be referenced. If validation was conducted for this study and not published elsewhere, detailed methods and results should be provided.<br>6.3: If the study involved linkage of databases, consider use of a flow diagram or other graphical display to demonstrate the data linkage process, including the number of individuals with linked data at each stage.	6.1.a: Describe the study entry criteria and the order in which these criteria were applied to identify the study population. Specify whether only users with a specific indication were included and whether patients were allowed to enter the study population once or if multiple entries were permitted. See explanatory document for guidance related to matched designs.	5
Variables				
7	Clearly define all outcomes, exposures, predictors, potential confounders, and effect modifiers. Give diagnostic criteria, if applicable.	7.1: A complete list of codes and algorithms used to classify exposures, outcomes, confounders, and effect modifiers should be provided. If these cannot be reported, an explanation should be provided.	7.1.a: Describe how the drug exposure definition was developed.<br>7.1.b: Specify the data sources from which drug exposure information for individuals was obtained.<br>7.1.c: Describe the time window(s) during which an individual is considered exposed to the drug(s). The rationale for selecting a particular time window should be provided. The extent of potential left truncation or left censoring should be specified.<br>7.1.d: Justify how events are attributed to current, prior, ever, or cumulative drug exposure.<br>7.1.e: When examining drug dose and risk attribution, describe how current, historical or time on therapy are considered.	5-6

			7.1.f: Use of any comparator groups should be outlined and justified.<br>7.1.g: Outline the approach used to handle individuals with more than one relevant drug exposure during the study period.	
Data sources/measurement				
8	For each variable of interest, give sources of data and details of methods of assessment (measurement). Describe comparability of assessment methods if there is more than one group.	—	8.a: Describe the healthcare system and mechanisms for generating the drug exposure records. Specify the care setting in which the drug(s) of interest was prescribed.	5-6
Bias				
9	Describe any efforts to address potential sources of bias.	—	—	NA
Study size				
10	Explain how the study size was arrived at.	—	—	NA
Quantitative variables				
11	Explain how quantitative variables were handled in the analyses. If applicable, describe which groupings were chosen, and why.	—	—	6-7
Statistical methods				
12	(a) Describe all statistical methods, including those used to control for confounding.<br>(b) Describe any methods used to examine subgroups and interactions.<br>(c) Explain how missing data were addressed.<br>(d) Cohort study—if applicable, explain how loss to follow-up was addressed. Case-control study—if applicable, explain how matching of cases and controls was addressed. Cross sectional study—if applicable, describe analytical methods taking account of sampling strategy.<br>(e) Describe any sensitivity analyses.	—	12.1.a: Describe the methods used to evaluate whether the assumptions have been met.<br>12.1.b: Describe and justify the use of multiple designs, design features, or analytical approaches.	6-7 Appendix 1
Data access and cleaning methods				
12	—	12.1: Authors should describe the extent to which the investigators had access to the database population used to create the study population.	—	

		12.2: Authors should provide information on the data cleaning methods used in the study.		
Linkage				
12	—	12.3: State whether the study included person level, institutional level, or other data linkage across two or more databases. The methods of linkage and methods of linkage quality evaluation should be provided.	—	NA
**Results**				
Participants				
13	(a) Report the numbers of individuals at each stage of the study (eg, numbers potentially eligible, examined for eligibility, confirmed eligible, included in the study, completing follow-up, and analysed).<br>(b) Give reasons for non-participation at each stage.<br>(c) Consider use of a flow diagram.	13.1: Describe in detail the selection of the individuals included in the study (that is, study population selection) including filtering based on data quality, data availability, and linkage. The selection of included individuals can be described in the text or by means of the study flow diagram.	—	7
Descriptive data				
14	(a) Give characteristics of study participants (eg, demographic, clinical, social) and information on exposures and potential confounders.<br>(b) Indicate the number of participants with missing data for each variable of interest.<br>(c) Cohort study—summarise follow-up time (eg, average and total amount).	—	—	7
Outcome data				
15	Cohort study—report numbers of outcome events or summary measures over time. Case-control study—report numbers in each exposure category, or summary measures of exposure. Cross sectional study—report numbers of outcome events or summary measures.	—	—	8
Main results				
16	(a) Give unadjusted estimates and, if applicable, confounder adjusted estimates and their precision (eg, 95%	—	—	8-9

	confidence intervals). Make clear which confounders were adjusted for and why they were included.<br>(b) Report category boundaries when continuous variables are categorised.<br>(c) If relevant, consider translating estimates of relative risk into absolute risk for a meaningful time period.			
Other analyses				
17	Report other analyses done—eg, analyses of subgroups and interactions, and sensitivity analyses.	—	—	NA
**Discussion**				
Key results				
18	Summarise key results with reference to study objectives.	—	—	9
Limitations				
19	Discuss limitations of the study, taking into account sources of potential bias or imprecision. Discuss both direction and magnitude of any potential bias.	19.1: Discuss the implications of using data that were not created or collected to answer the specific research question(s). Include discussion of misclassification bias, unmeasured confounding, missing data, and changing eligibility over time, as they pertain to the study being reported.	19.1.a: Describe the degree to which the chosen database(s) adequately captures the drug exposure(s) of interest.	11
Interpretation				
20	Give a cautious overall interpretation of results considering objectives, limitations, multiplicity of analyses, results from similar studies, and other relevant evidence.	—	20.a: Discuss the potential for confounding by indication, contraindication or disease severity or selection bias (healthy adherer/sick stopper) as alternative explanations for the study findings when relevant. **[A: Original text indicated this item was RECORD (ie, not RECORD-PE)?]**	9-10
Generalisability				
21	Discuss the generalisability (external validity) of the study results.	—	—	11
**Other information**				
Funding				
22	Give the source of funding and the role of the funders for the present study and, if applicable, for the original	—	—	12

	study on which the present article is based.			
Accessibility of protocol, raw data, and programming code				
22	—	22.1: Authors should provide information on how to access any supplemental information such as the study protocol, raw data, or programming code.	—	12

RECORD=reporting of studies conducted using observational routinely collected data; RECORD-PE=RECORD for pharmacoepidemiological research; STROBE=strengthening the reporting of observational studies in epidemiology.

REFERENCE:
Langan SM, Schmidt S, Wing K, Ehrenstein V, Nicholls S, Filion K, Klungel O, Petersen I, Sorensen H, Guttmann A, Harron K, Hemkens L, Moher D, Schneeweiss S, Smeeth L, Sturkenboom M, von Elm E, Wang S, Benchimol EI. The REporting of studies Conducted using Observational Routinely-collected health Data (RECORD) Statement for Pharmacoepidemiology (RECORD-PE). *BMJ* 2018; 363: k3532.

Table S2. List of medicines monitored by the Victorian Prescription Drug Monitoring Program

Generic name	ATC code
**OPIOIDS**	
Buprenorphine	N02AE01
**Buprenorphine/naloxone**	N07BC51
Codeine	R05DA04
Codeine combinations	
Aspirin/codeine	N02AJ07
Ibuprofen/codeine	N02AJ08
Paracetamol/codeine	N02AA59, N02AJ06
Paracetamol/codeine/doxylamine	N02AA59, N02AJ06
Fentanyl	N02AB03, N01AH01
Hydromorphone	N02AA03
Methadone	N02AC52, N07BC02
Morphine	N02AA01
Oxycodone	N02AA05
Oxycodone/naloxone	N02AA55
Pethidine	N02AB02
Tapentadol	N02AX06
**BENZODIAZEPINES**	
Alprazolam	N05BA12
Bromazepam	N05BA08
Clobazam	N05BA09
Clonazepam	N03AE01
Diazepam	N05BA01
Flunitrazepam	N05CD03
Lorazepam	N05BA06
Midazolam	N05CD08
Nitrazepam	N05CD02
Oxazepam	N05BA04
Temazepam	N05CD07
**Z-DRUGS**	
Zolpidem	N05CF02
Zopiclone	N05CF01
**STIMULANTS**	
Dexamphetamine	N06BA02
Lisdexamphetamine	N06BA12
Methylphenidate	N06BA04

**CANNABINOIDS**	
Cannabidiol	N03AX24
Cannabidiol/tetrahydrocannabinol	N02BG10
**OTHER HIGH-RISK MEDICINES**	
Ketamine	N01AX03
Quetiapine	N05AH04

ATC: Anatomical Therapeutic Chemical (ATC) classification system

Table S3. List of other psychotropic medicines not monitored by the Victorian Prescription Drug Monitoring Program

Generic name	ATC code
**Antidepressants**	
**Selective serotonin reuptake inhibitors**	
Citalopram	N06AB04
Escitalopram	N06AB10
Fluoxetine	N06AB03
Fluvoxamine	N06AB08
Paroxetine	N06AB05
Sertraline	N06AB06
**Tricyclic Antidepressants**	
Amitriptyline	N06AA09
Clomipramine	N06AA04
Dosulepin	N06AA16
Doxepin	N06AA12
Imipramine	N06AA02
Nortriptyline	N06AA10
**Serotonin-norepinephrine reuptake inhibitors**	
Desvenlafaxine	N06AX23
Duloxetine	N06AX21
Venlafaxine	N06AX16
**Other antidepressants**	
Agomelatine	N06AX22
Mianserin	N06AX03
Mirtazapine	N06AX11
Moclobemide	N06AG02
Phenelzine	N06AF03
Reboxetine	N06AX18
Tranylcypromine	N06AF04
Vortioxetine	N06AX26
**Antipsychotics**	
Amisulpride	N05AL05
Ariprazole	N05AX12
Asenapine	N05AH05
Brexipiprazole	N05AX16
Cariprazine	N05AX15
Chlorpromazine	N05AA01
Clozapine	N05AH02
Droperidol	N05AD08
Flupentixol	N05AF01
Haloperidol	N05AD01
Lurasidone	N05AE05
Olanzapine	N05AH03
Paliperidone	N05AX13
Periciazine	N05AC01

Risperidone	N05AX08
Ziprasidone	N05AE04
Zuclopenthixol	N05AF05
**Medicines for bipolar disorder**	
Carbamazepine	N03AF01
Lamotrigine	N03AX09
Lithium	N05AN01
Valproate	N03AG
**Other medicines for sleep**	
Buspirone	N05BE01
Lemorexant	N05CM21
Melatonin	N05CH01
Suvorexant	N05CM19
**Gabapentinoids**	
Gabapentin	N02BF01
Pregabalin	N02BF02
**Other non-monitored opioids**	
Tramadol	N02AX02
Tramadol combination with paracetamol	N02AJ13

ATC: Anatomical Therapeutic Chemical (ATC) classification system

Table S4.1 List of mental health diagnosis codes

SNOMED Code	SNOMED Text
67195008	ACUTE STRESS DISORDER
17226007	ADJUSTMENT DISORDER
47372000	ADJUSTMENT DISORDER WITH ANXIOUS MOOD
57194009	ADJUSTMENT DISORDER WITH DEPRESSED MOOD
70691001	AGORAPHOBIA
48694002	ANXIETY
247808006	ANXIETY ABOUT BODY FUNCTION OR HEALTH
260994008	BIPOLAR
13746004	BIPOLAR DISORDER
371596008	BIPOLAR I DISORDER
83225003	BIPOLAR II DISORDER
274952002	BORDERLINE SCHIZOPHRENIA
111489007	BREATHING-RELATED SLEEP DISORDER
191708009	CHRONIC ANXIETY
83746006	CHRONIC SCHIZOPHRENIA
19887002	CLAUSTROPHOBIA
12479006	COMPULSIVE BEHAVIOUR
18085000	COMPULSIVE GAMBLING
473456000	COMPULSIVE PERSONALITY DISORDER
76105009	CYCLOTHYMIA
48500005	DELUSIONAL DISORDER
2073000	DELUSIONS
366979004	DEPRESSED MOOD
35489007	DEPRESSION
301345002	DIFFICULTY SLEEPING
78667006	DYSTHYMIA
309838005	EMOTIONAL UPSET
300706003	ENDOGENOUS DEPRESSION
230490003	EXCESSIVE DAY AND NIGHT-TIME SLEEPINESS
247853008	FEAR OF FLYING
34563004	FEAR OF GETTING CANCER
102931001	FEAR OF HEART ATTACK
191733007	FEAR OF PREGNANCY
247854002	FLYING PHOBIA
66347000	IMPULSE CONTROL DISORDER
59050008	INITIAL INSOMNIA
193462001	INSOMNIA
69482004	KORSAKOFF'S PSYCHOSIS
162204000	LATE INSOMNIA
370143000	MAJOR DEPRESSIVE DISORDER
67233009	MIDDLE INSOMNIA
231504006	MIXED ANXIETY AND DEPRESSIVE DISORDER
46206005	MOOD DISORDER
231501003	NEEDLE PHOBIA

191736004	OBSESSIVE-COMPULSIVE DISORDER
112091009	OBSESSIVE-COMPULSIVE PERSONALITY TRAIT
10586006	OCCUPATION-RELATED STRESS DISORDER
79823003	PANIC
225624000	PANIC ATTACK
371631005	PANIC DISORDER
64905009	PARANOID SCHIZOPHRENIA
279622009	PERFORMANCE ANXIETY
386808001	PHOBIA
386810004	PHOBIC DISORDER
58535001	PHYSICAL & EMOTIONAL EXHAUSTION STATE
47505003	POST-TRAUMATIC STRESS DISORDER
58703003	POSTPARTUM DEPRESSION
69322001	PSYCHOTIC DISORDER
87414006	REACTIVE DEPRESSION (SITUATIONAL)
68890003	SCHIZOAFFECTIVE DISORDER
38368003	SCHIZOAFFECTIVE DISORDER, BIPOLAR TYPE
58214004	SCHIZOPHRENIA
191526005	SCHIZOPHRENIC DISORDERS
248061004	SELF-HARM
130968006	SELF-MUTILATION
126943008	SEPARATION ANXIETY
11806006	SEPARATION ANXIETY DISORDER OF CHILDHOOD
130989002	SLEEP DEPRIVATION
39898005	SLEEP DISORDER
277180005	SLEEP PARALYSIS
164773008	SLEEP STUDIES
89675003	SLEEP TERROR DISORDER
80495009	SLEEP WALKING
25501002	SOCIAL PHOBIA
73595000	STRESS
276074009	STRESS AT WORK
267073005	SUICIDAL
6471006	SUICIDAL THOUGHTS
82313006	SUICIDE ATTEMPT
102497008	SYMPTOMS OF STRESS
248265004	WORK STRESS

SNOMED: Systemized Nomenclature of Medicine.

Table S4.2 List of substance use disorder diagnosis codes

SNOMED Code	SNOMED Text
31684002	BUPRENORPHINE
425741009	BUPRENORPHINE+NALOXONE
398705004	CANNABIS
703848005	CANNABIS WITHDRAWAL
78267003	COCAINE ABUSE
31956009	COCAINE DEPENDENCE
219006	CURRENT DRINKER
6525002	DEPENDENT DRUG ABUSE
228326007	DRINKING BINGE
26416006	DRUG ABUSE
228373001	DRUG ADDICT
191816009	DRUG DEPENDENCE
55680006	DRUG OVERDOSE
441668002	DRUG SEEKING BEHAVIOUR
363101005	DRUG WITHDRAWAL
398624005	ETHYL ALCOHOL
231477003	HEROIN DEPENDENCE
228388006	INTRAVENOUS DRUG USER
231479000	MORPHINE DEPENDENCE
360204007	OPIATE
5602001	OPIOID ABUSE
75544000	OPIOID DEPENDENCE
242253008	OVERDOSE OF OPIATE
295124009	PARACETAMOL OVERDOSE
284591009	PERSISTENT ALCOHOL ABUSE
228372006	POLY-DRUG MISUSER
445273005	POLYSUBSTANCE ABUSE
228281002	PROBLEM DRINKER
91388009	PSYCHOACTIVE SUBSTANCE ABUSE
424848002	RECREATIONAL DRUG USER
64386003	SEDATIVE ABUSE
66214007	SUBSTANCE ABUSE
424589009	SUBSTANCE USE TREATMENT: CESSATION

SNOMED: Systemized Nomenclature of Medicine.

Table S4.3 List of headache/migraine diagnosis codes

SNOMED Code	SNOMED Text
83351003	BASILAR MIGRAINE
279016001	CERVICOGENIC HEADACHE
431237007	CHRONIC HEADACHE DISORDER
230477005	CHRONIC POST-TRAUMATIC HEADACHE
230471006	CHRONIC TENSION-TYPE HEADACHE
193031009	CLUSTER HEADACHE
193039006	COMPLICATED MIGRAINE
571000119103	DAILY HEADACHE
43242008	DRUG WITHDRAWAL HEADACHE
267096005	FRONTAL HEADACHE
25064002	HEADACHE
59292006	HEMIPLEGIC MIGRAINE
37796009	MIGRAINE
230465000	MIGRAINE AURA WITHOUT HEADACHE
408381007	MIGRAINE PROPHYLAXIS
4473006	MIGRAINE WITH AURA
56097005	MIGRAINE WITHOUT AURA
398126006	MUSCULAR HEADACHE
121021000119105	NEW DAILY PERSISTENT HEADACHE
95655001	OPHTHALMIC MIGRAINE
49605003	OPHTHALMOPLEGIC MIGRAINE
66551002	PSYCHOGENIC HEADACHE
398057008	TENSION-TYPE HEADACHE

SNOMED: Systemized Nomenclature of Medicine.

Table S4.4 List of chronic pain diagnosis codes

SNOMED Code	SNOMED Text
9631008	ANKYLOSING SPONDYLITIS
111985007	CHRONIC ABDOMINAL PAIN
134407002	CHRONIC BACK PAIN
431237007	CHRONIC HEADACHE DISORDER
278860009	CHRONIC LOWER BACK PAIN
1121000119107	CHRONIC NECK PAIN
82423001	CHRONIC PAIN
432615008	CHRONIC PAIN IN FACE
373621006	CHRONIC PAIN SYNDROME
237067000	CHRONIC PELVIC PAIN OF FEMALE
230477005	CHRONIC POST-TRAUMATIC HEADACHE
128200000	COMPLEX REGIONAL PAIN SYNDROME
50642008	COMPLEX REGIONAL PAIN SYNDROME TYPE I
203082005	FIBROMYALGIA
56557000	FIBROSITIS
410795001	JUVENILE RHEUMATOID ARTHRITIS
50442003	PALINDROMIC RHEUMATISM
396332003	RHEUMATISM
69896004	RHEUMATOID ARTHRITIS
239792003	SERONEGATIVE RHEUMATOID ARTHRITIS
275488008	SORE THROAT - CHRONIC

SNOMED: Systemized Nomenclature of Medicine.

Appendix 1: Model specifications

Changes were evaluated with segmented linear regression models, using the ordinary least squares method with Newey–West standard errors adjustment. Autocorrelation was assessed using the Cumby–Huizinga test, which evaluates the null hypothesis that the error term follows a moving average process up to order q. The test was implemented in Stata 17 using the ‘actest’ command to account for conditional heteroskedasticity, with autocorrelation examined up to 12 lags. Based on these results, lagged terms (up to three lags) were included in the models to account for autocorrelation (1). To address potential seasonality, we incorporated sine and/or cosine terms for each calendar month (2). After fitting the initial models with these terms, the final model retained the sin 2, cos 2, sin 4, and cos 5 terms, which were statistically significant ($p < 0.05$). As Victorian Coronavirus disease (COVID-19) related lockdown periods from May 2020 to October 2021 could impact the seeking and delivery of health care (3,4), our model was also adjusted to account for the number of days under lockdown at each monthly data point. Differential step/level and changes in the rate of seeing multiple prescribers between people prescribed monitored and non-monitored medicines were reported, along with 95% confidence intervals (CI).

References:

1. Linden A. Conducting Interrupted Time-series Analysis for Single- and Multiple-group Comparisons. The Stata Journal. 2015;15(2):480-500.
2. Jebb AT, Tay L, Wang W, Huang Q. Time series analysis for psychological research: examining and forecasting change. Frontiers in psychology. 2015;6:727.
3. Bell JS, Reynolds L, Freeman C, Jackson JK. Strategies to promote access to medications during the COVID-19 pandemic. The Australian Journal of General Practice. 2020;49(8):530-2.
4. Jung M, Lukose D, Nielsen S, Bell JS, Webb GI, Ilomaki J. COVID-19 restrictions and the incidence and prevalence of prescription opioid use in Australia - a nationwide study. British Journal of Clinical Pharmacology. 2023;89(2):914-20.
